# Genome-wide characterization of the WRKY gene family in cultivated strawberry (*Fragaria* × *ananassa* Duch.) and the importance of several group III members in continuous cropping

**DOI:** 10.1038/s41598-019-44479-7

**Published:** 2019-06-10

**Authors:** Peng Chen, Qi-zhi Liu

**Affiliations:** Laboratory of Entomology and Nematology, College of Plant Protection, China Agricultural University, Beijing, 100193 China

**Keywords:** Transcriptomics, Plant stress responses

## Abstract

WRKY transcription factors play important roles in many plant developmental processes and adaptation to the environment. However, little knowledge is available about the WRKY gene family in cultivated strawberry (*Fragaria* × *ananassa* Duch.), an important soft fruit worldwide. In this study, a total of 47 WRKY gene members were identified and renamed on the basis of their order on the chromosomes. According to their evolutionary events and conserved structure, the 47 *FaWRKYs* were divided into three major groups with several subgroups. A *cis*-element analysis showed that all *FaWRKYs* possessed at least one stress response-related *cis*-element. Comprehensive analysis, including phylogenetic analysis and expression profiling, based on real-time qPCR analysis in root, stem, leaf and fruit was performed on group III *FaWRKY* genes. The phylogenetic tree of the WRKY III genes in cultivated strawberry, wild Strawberry, Arabidopsis, tomato, and rice was divided into five clades. Additionally, the expression profiles of the *FaWRKY* genes in response to continuous cropping were further investigated based on RNA-seq data. *FaWRKY25*, *FaWRKY32*, and *FaWRKY45*, which are group III Fa*WRKY* genes, were upregulated after continuous cropping. The level of reactive oxygen species (ROS) and the expression levels of *PR1* and *peroxidase* were higher in continuous cropping (CC) than in non-continuous cropping (NCC). The results indicated that group III *FaWRKYs* might play an important role in continuous cropping. These results provide a foundation for genetic improvements for continuous cropping tolerance in cultivated strawberry.

## Introduction

Plants are frequently faced with multiple environmental changes, including biotic and abiotic stresses. Biotic stresses include insect pests, fungi, bacteria and viruses, and abiotic stresses include heat, cold, drought, salinity and wounding^1–4^. Being sessile organisms, plants sense these stresses through complex signal transduction networks and have evolved specific defensive strategies to adapt to environmental fluctuations during their long evolutionary process^5,6^. Transcriptional regulation is a major mechanism for organisms to regulate their gene expression in response to physiological or environmental stimuli^6^. Families of transcription factors regulate gene expression in cells or organisms. They often harbor a DNA-binding region and play an essential role in activating or repressing the rate of transcription of specific target genes^7^.

The WRKY family of transcription factors was first isolated from plants. Over twenty years ago, Ishiguro and Nakamura identified the first WRKY protein (SPF1) from sweet potato^8^. *WRKY* genes form a family of regulatory genes, and the name is derived from the most prominent feature in their WRKY domain, which is composed of a highly conserved region consisting of 60 amino acid residues^9^. The WRKY amino acid sequence shows high binding affinity to a conserved cognate binding site designated the W box (C/TTGACT/C)^9,10^.

Numerous *WRKY* genes have since been identified from many plant species, including more than 70 WRKY members in *Arabidopsis thaliana*^11,12^, 81 WRKY members in *Solanum lycopersicum*^5^, 102 and 98 WRKY members in *Oryza sativa* L. ssp. *indica* and L. ssp. *japonica*^13^, 59 WRKY members in *Fragaria vesca*^14^, and 54 WRKY members in *Ananas comosus*^15^. The WRKY protein family can be classified into three major distinct groups (Groups I, II, and III) depending on the number of WRKY domains and the type of zinc finger motif^9^. WRKY group I proteins contain two WRKY domains and a C2H2 zinc finger motif; group II proteins encompassing subgroups (II a–e) have one WRKY domain containing the same C2H2 zinc finger motif; and group III proteins have one WRKY domain containing the specific C2HC zinc finger motif^9^.

Cultivated strawberry (*Fragaria* × *ananassa* Duch.) is an octoploid species (2n = 8x = 56) and is an important soft fruit worldwide^16^. Strawberries are famous for their delicious taste, attractive appearance and abundant phytochemical compounds^17^. Zhou *et al*. performed a genome-wide analysis of wild strawberry (*Fragaria vesca*) WRKY genes, identified 59 *FvWRKY* genes in wild strawberry, and analyzed their expression in different fruit developmental stages^14^. However, little knowledge is available about the WRKY gene family in cultivated strawberry.

In this study, we identified 47 cultivated strawberry *WRKY* gene members and classified them into three major groups with several subgroups. A comprehensive analysis including phylogenetic relationships, multiple sequence alignment, and stress-related *cis-*element analysis was further performed. The expression patterns of group III *FaWRKY* genes in different organs, including root, stem, leaf and fruit, were analyzed by real-time quantitative RT-PCR. Global expression analysis of *WRKY* genes was performed under continuous cropping conditions based on RNA-seq data.

## Results

### Identification and analysis of FaWRKY proteins

**Figure 1:**
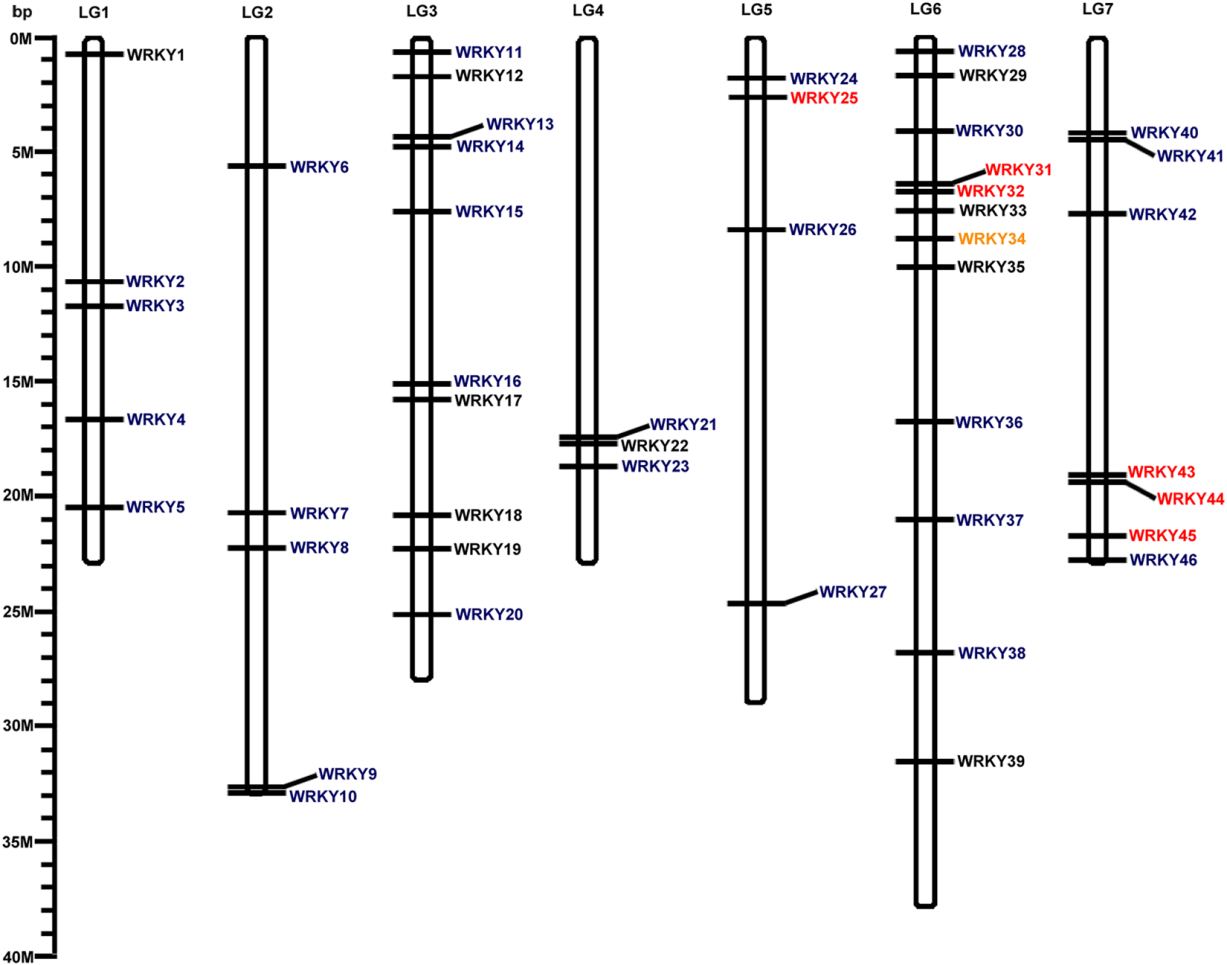
Chromosomal distributions of *FaWRKY* genes. Chromosome numbers are showed at the top of each chromosome and the approximate size in megabases (Mb) provided at the left side. The names of each *FaWRKY* gene showed on the right side of each chromosome. Group I members are indicated in black font; group II members are indicated in blue font; group III members are indicated in red font. *FaWRKY34* in orange font not belongs to any group. Unmapped WRKY genes (*FaWRKY47*) are not shown.

A total of 52 candidate WRKY proteins were obtained from PlantTFDB (http://planttfdb.cbi.pku.edu.cn/) in *Fragaria x ananassa* Duch. The annotation of these putative genes was further checked using BLASTP analysis in NCBI (https://www.ncbi.nlm.nih.gov). Three erroneously predicted WRKY proteins (FANhyb_rscf00000209.1.g00016.1, FANhyb_rscf00000217.1.g00006.1 and FANhyb_rscf00003540.1.g00001.1) were manually curated, and two redundant sequences (FANhyb_icon00001572_a.1.g00001.1 and FANhyb_icon00043401_a.1.g00001.1) were then removed. Finally, 47 sequences were confirmed and annotated as being cultivated strawberry *WRKY* genes. To better reflect the appropriate function, we named each *FaWRKY* based on their order on the chromosomes, and hit them to the similar individual *AtWRKYs* (Fig. 1, Table 1). Gene names, gene IDs, chromosomal locations, genomic locations, homologous genes, hit IDs and blast E-values are listed in Table 1.

**Table 1:** List of *WRKY* genes identified in *Fragaria* × *ananassa* Duch.

	Gene name	Gene ID	Chr	Genomic Location	Orthologous gene	Hit ID	E-value
1	*FaWRKY1*	FANhyb_rscf00000014.1.g00012.1	LG1	NC_020491.1 (870919–874229)	*AtWRKY32*	AT4G30935.1	1e-103
2	*FaWRKY2*	FANhyb_icon00000687_a.1.g00001.1	LG1	NC_020491.1 (10577850–10579374)	*AtWRKY15*	AT2G23320.1	2e-73
3	*FaWRKY3*	FANhyb_rscf00000204.1.g00010.1	LG1	NC_020491.1 (11770138–11773994)	*AtWRKY13*	AT4G39410.1	4e-19
4	*FaWRKY4*	FANhyb_rscf00000130.1.g00007.1	LG1	NC_020491.1 (16629836–16633210)	*AtWRKY47*	AT4G01720.1	5e-56
5	*FaWRKY5*	FANhyb_rscf00000722.1.g00001.1	LG1	NC_020491.1 (20465509–20467726)	*AtWRKY23*	AT2G47260.1	3e-45
6	*FaWRKY6*	FANhyb_rscf00000917.1.g00008.1	LG2	NC_020492.1 (5696899–5699900)	*AtWRKY21*	AT2G30590.1	1e-81
7	*FaWRKY7*	FANhyb_icon00001105_a.1.g00001.1	LG2	NC_020492.1 (20833008–20836027)	*AtWRKY7*	AT4G24240.1	4e-65
8	*FaWRKY8*	FANhyb_icon00010025_a.1.g00001.1	LG2	NC_020492.1 (22347375–22349507)	*AtWRKY11*	AT4G31550.1	1e-52
9	*FaWRKY9*	FANhyb_rscf00000031.1.g00036.1	LG2	NC_020492.1 (33088502–33091155)	*AtWRKY40*	AT1G80840.1	5e-58
10	*FaWRKY10*	FANhyb_rscf00000031.1.g00037.1	LG2	NC_020492.1 (33093532–33095374)	*AtWRKY40*	AT1G80840.1	5e-58
11	*FaWRKY11*	FANhyb_rscf00000892.1.g00009.1	LG3	NC_020493.1 (635027–637933)	*AtWRKY31*	AT1G62300.1	1e-123
12	*FaWRKY12*	FANhyb_rscf00005774.1.g00001.1	LG3	NC_020493.1 (1716020–1719529)	*AtWRKY1*	AT2G04880.2	1e-83
13	*FaWRKY13*	FANhyb_icon00024544_a.1.g00001.1	LG3	NC_020493.1 (4533799..4536252)	*AtWRKY71*	AT1G29860.1	3e-25
14	*FaWRKY14*	FANhyb_rscf00000094.1.g00017.1	LG3	NC_020493.1 (4856445–4859544)	*AtWRKY14*	AT1G30650.1	8e-69
15	*FaWRKY15*	FANhyb_icon00019813_a.1.g00001.1	LG3	NC_020493.1 (7633622–7635218)	*AtWRKY65*	AT1G29280.1	6e-36
16	*FaWRKY16*	FANhyb_rscf00000405.1.g00006.1	LG3	NC_020493.1 (15053416..15056443)	*AtWRKY31*	AT1G62300.1	2e-84
17	*FaWRKY17*	FANhyb_rscf00000296.1.g00003.1	LG3	NC_020493.1 (15885293–15890393)	*AtWRKY20*	AT4G26640.1	1e-166
18	*FaWRKY18*	FANhyb_rscf00001700.1.g00004.1	LG3	NC_020493.1 (20956047–20959174)	*AtWRKY25*	AT2G38470.1	2e-35
19	*FaWRKY19*	FANhyb_rscf00000701.1.g00002.1	LG3	NC_020493.1 (22341618–22346373)	*AtWRKY2*	AT5G56270.1	0.0
20	*FaWRKY20*	FANhyb_icon00033462_a.1.g00001.1	LG3	NC_020493.1 (25007887–25010163)	*AtWRKY48*	AT5G49520.1	1e-25
21	*FaWRKY21*	FANhyb_icon00011092_a.1.g00001.1	LG4	NC_020494.1 (17481528–17484817)	*AtWRKY57*	AT1G69310.2	3e-23
22	*FaWRKY22*	FANhyb_rscf00000106.1.g00008.1	LG4	NC_020494.1 (17623403–17626444)	*AtWRKY3*	AT2G03340.1	1e-138
23	*FaWRKY23*	FANhyb_rscf00000290.1.g00001.1	LG4	NC_020494.1 (18215850–18219380)	*AtWRKY72*	AT5G15130.1	2e-71
24	*FaWRKY24*	FANhyb_rscf00000119.1.g00009.1	LG5	NC_020495.1 (1863533–1865479)	*AtWRKY29*	AT4G23550.1	2e-43
25	*FaWRKY25*	FANhyb_rscf00001973.1.g00002.1	LG5	NC_020495.1 (2523342–2525716)	*AtWRKY53*	AT4G23810.1	6e-55
26	*FaWRKY26*	FANhyb_rscf00002639.1.g00001.1	LG5	NC_020495.1 (8486170–8489600)	*AtWRKY72*	AT5G15130.1	9e-71
27	*FaWRKY27*	FANhyb_rscf00006696.1.g00001.1	LG5	NC_020495.1 (24702940–24704404)	*AtWRKY35*	AT2G34830.1	1e-35
28	*FaWRKY28*	FANhyb_icon00011738_a.1.g00001.1	LG6	NC_020496.1 (642733–645019)	*AtWRKY74*	AT5G28650.1	5e-33
29	*FaWRKY29*	FANhyb_icon00000768_a.1.g00001.1	LG6	NC_020496.1 (1832751–1837559)	*AtWRKY34*	AT4G26440.1	9e-59
30	*FaWRKY30*	FANhyb_rscf00000319.1.g00011.1	LG6	NC_020496.1 (4272821–4275209)	*AtWRKY72*	AT5G15130.1	3e-70
31	*FaWRKY31*	FANhyb_icon00015805_a.1.g00001.1	LG6	NC_020496.1 (6539328–6542693)	*AtWRKY70*	AT3G56400.1	2e-29
32	*FaWRKY32*	FANhyb_rscf00004695.1.g00001.1	LG6	NC_020496.1 (6567849–6569763)	*AtWRKY70*	AT3G56400.1	3e-39
33	*FaWRKY33*	FANhyb_rscf00001513.1.g00003.1	LG6	NC_020496.1 (7059890–7062489)	*AtWRKY33*	AT2G38470.1	1e-108
34	*FaWRKY34*	FANhyb_icon00000456_a.1.g00001.1	LG6	NC_020496.1 (8879460–8882223)	*AtWRKY49*	AT5G43290.1	2e-28
35	*FaWRKY35*	FANhyb_rscf00001029.1.g00002.1	LG6	NC_020496.1 (10015991–10020299)	*AtWRKY44*	AT2G37260.1	1e-110
36	*FaWRKY36*	FANhyb_rscf00001608.1.g00001.1	LG6	NC_020496.1 (16823225–16825479)	*AtWRKY40*	AT1G80840.1	8e-90
37	*FaWRKY37*	FANhyb_rscf00000148.1.g00011.1	LG6	NC_020496.1 (21039097–21040162)	*AtWRKY50*	AT5G26170.1	3e-45
38	*FaWRKY38*	FANhyb_rscf00000625.1.g00005.1	LG6	NC_020496.1 (26896885–26900342)	*AtWRKY71*	AT5G46350.1	2e-21
39	*FaWRKY39*	FANhyb_rscf00000848.1.g00005.1	LG6	NC_020496.1 (31544425–31547916)	*AtWRKY3*	AT2G03340.1	1e-105
40	*FaWRKY40*	FANhyb_rscf00000687.1.g00006.1	LG7	NC_020497.1 (4326075–4327864)	*AtWRKY43*	AT2G46130.1	1e-55
41	*FaWRKY41*	FANhyb_icon00011989_a.1.g00001.1	LG7	NC_020497.1 (4348088–4349716)	*AtWRKY22*	AT4G01250.1	5e-19
42	*FaWRKY42*	FANhyb_rscf00001075.1.g00001.1	LG7	NC_020497.1 (7738940–7741851)	*AtWRKY12*	AT2G44745.1	5e-56
43	*FaWRKY43*	FANhyb_rscf00000201.1.g00016.1	LG7	NC_020497.1 (19102161–19104605)	*AtWRKY55*	AT2G40740.1	1e-54
44	*FaWRKY44*	FANhyb_icon00007002_a.1.g00001.1	LG7	NC_020497.1 (19104994–19107275)	*AtWRKY70*	AT3G56400.1	2e-29
45	*FaWRKY45*	FANhyb_rscf00000702.1.g00006.1	LG7	NC_020497.1 (21782499–21784846)	*AtWRKY41*	AT4G23810.1	4e-54
46	*FaWRKY46*	FANhyb_icon00006561_a.1.g00001.1	LG7	NC_020497.1 (22089165–22090972)	*AtWRKY27*	AT5G52830.1	3e-26
47	*FaWRKY47*	FANhyb_rscf00001565.1.g00004.1	Unplaced Scaffold	NW_004443448.1 (1339517..1342239)	*AtWRKY75*	AT5G13080.1	7e-21

### Phylogenetic analysis and multiple sequence alignment

**Figure 2:**
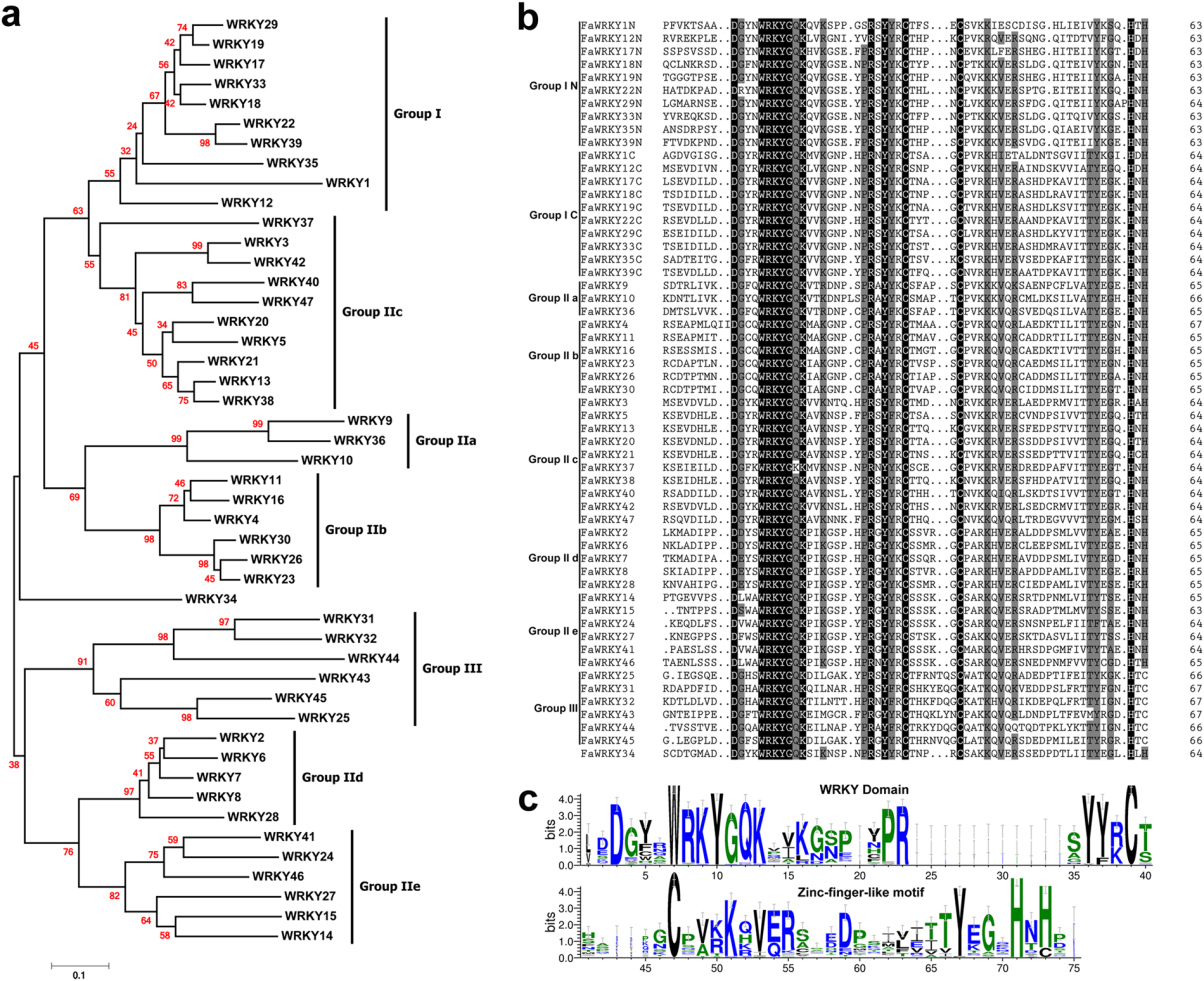
The phylogenetic tree, Multiple sequence and domain alignment of FaWRKY proteins. (**a**) The phylogenetic tree representing relationships among FaWRKY proteins using the neighbor-joining method. The bootstrap values with 1,000 bootstrap replications are shown above the branches. The tree was divided into three major groups (I, II and III) with five subgroups (II a-e). (**b**) Multiple sequence alignment of FaWRKY domain amino acid sequences. Groups I N and I C indicate the N-terminus and C-terminus of the WRKY domain of a group I WRKY protein. (**c**) Domains of FaWRKY proteins.

To explore the phylogenetic relationship of the FaWRKY proteins, we constructed a phylogenetic tree of 47 FaWRKY proteins using MEGA 7.0. The phylogenetic analysis showed that the cultivated strawberry WRKY proteins could be divided into three major groups (groups I, II and III) with five subgroups (II a, II b, II c, II d, and II e) corresponding to the AtWRKY proteins^9^ (Fig. 2a). Among the 47 FaWRKY proteins, 10 belong to group I; 31 belong to group II, including 3 to group II a, 6 to group II b, 10 to group II c, 5 to group II d, and 7 to group II e; and 6 belong to group III. However, FaWRKY34 could not be clustered into any group.

Multiple sequence alignment of the WRKY domains, which span approximately 60 amino acids, revealed that the conserved amino acid sequences were defined by WRKYGQ(K)K at the N-terminal end, together with a zinc finger-like motif at the C-terminal end (Fig. 2b,c). The domain number, conserved heptapeptides and zinc finger types of each group are shown in Table 2. The group I FaWRKY proteins contained two WRKY domains (WRKYGQK) and C2H2-type zinc finger motifs, group IN and group IC. Groups II and III contained one WRKY domain and zinc finger motif. Most members of group II (a, b, c, d, e), except for FaWRKY50, which was defined by WRKYGKK and C2H2-type zinc finger motifs, were defined by WRKYGQK and C2H2-type zinc finger motifs. All six members in group III contain the conserved WRKYGQK and the special C2HC-type zinc fingers.

**Table 2:** WRKY domains of FaWRKYs.

Group	Subgroup	Gene number	Domain number	Conserved heptapeptide	Zinc-finger type
I		10	2	WRKYGQK/WRKYGQK	C2H2
II	II a	3	1	WRKYGQK	C2H2
	II b	6	1	WRKYGQK	C2H2
	II c	10	1	WRKYGQ(K)K	C2H2
	II d	5	1	WRKYGQK	C2H2
	II e	6	1	WRKYGQK	C2H2
III		6	1	WRKYGQK	C2HC
Other (*FaWRKY34*)		1	1	WRKYGQK	C2H2

### Stress-related *cis*-elements in *FaWRKY* promoter regions

**Figure 3:**
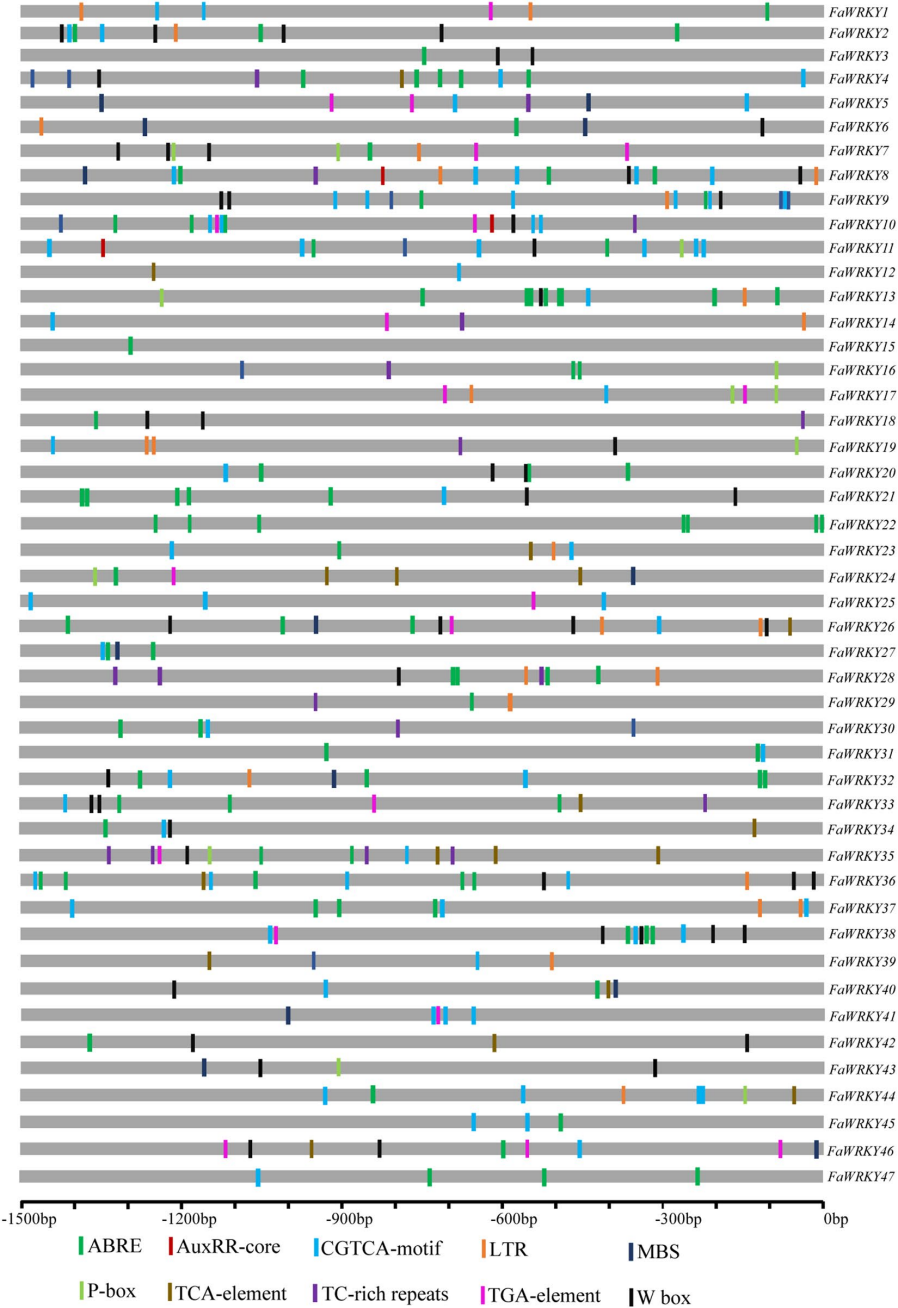
Predicted ***cis***-elements in *FaWRKY* promoters. Promoter sequences (−1500 bp) of 47 *FaWRKY* genes were analyzed by PlantCARE.

Plants use complex defense signaling pathways, including stress-related *cis*-elements, to regulate their adaptation to ever-changing stresses^18^.The WRKY TFs could regulate gene expression by binding to their *cis-*elements during stress responses. To further determine if WRKY TFs engage in potential regulatory mechanisms during stress responses, the promoter regions, sequences of approximately 1.5-kb upstream from the translation start sites, were submitted into PlantCARE to identify the *cis*-elements. In this study, ten stress response elements, including ABRE (*cis*-acting element involved in abscisic acid responsiveness), AuxRR-core (*cis*-acting regulatory element involved in auxin responsiveness), CGTCA-motif (*cis*-acting regulatory element involved in MeJA responsiveness), LTR (*cis*-acting element involved in low-temperature responsiveness), MBS (MYB transcription factor binding site involved in drought inducibility), P-box (gibberellin-responsive element), TCA-element (*cis*-acting element involved in salicylic acid responsiveness), TC-rich repeats (*cis*-acting element involved in defense and stress responsiveness), TGA-element (auxin-responsive element) and W-box (WRKY transcription factor binding site in defense responses), were analyzed, and the data are displayed in Fig. 3.

In this study, all FaWRKY TFs had at least 1 stress response-related *cis*-element. The elements associated with hormone regulation, including ABRE, AuxRR-core, CGTCA motif, P-box, TCA-element, and TGA-element, were identified in many *FaWRKY* promoter regions. In total, 38 *FaWRKYs* (80.8%) had one or more ABRE, suggesting a potential abscisic acid response under stress conditions. One or more CGTCA motifs, which are involved in MeJA responsiveness, existed in 33 *FaWRKYs* (70.2%). TCA-element, TGA-element, P-box and AuxRR-core were located in 14, 14, 10 and 3 *FaWRKYs*, respectively.

*Cis*-elements related to other stresses were found in several *FaWRKY* promoter regions. For example, 26 *FaWRKYs* contained one or more W-box elements in their promoter regions. In addition, several LTR, MBS and TC-rich repeats, which are involved in low temperature, drought inducibility and defense responsiveness, were also found in many *FaWRKYs*.

### Evolutionary analysis of *WRKY* group III TFs in strawberry and several different species

**Figure 4:**
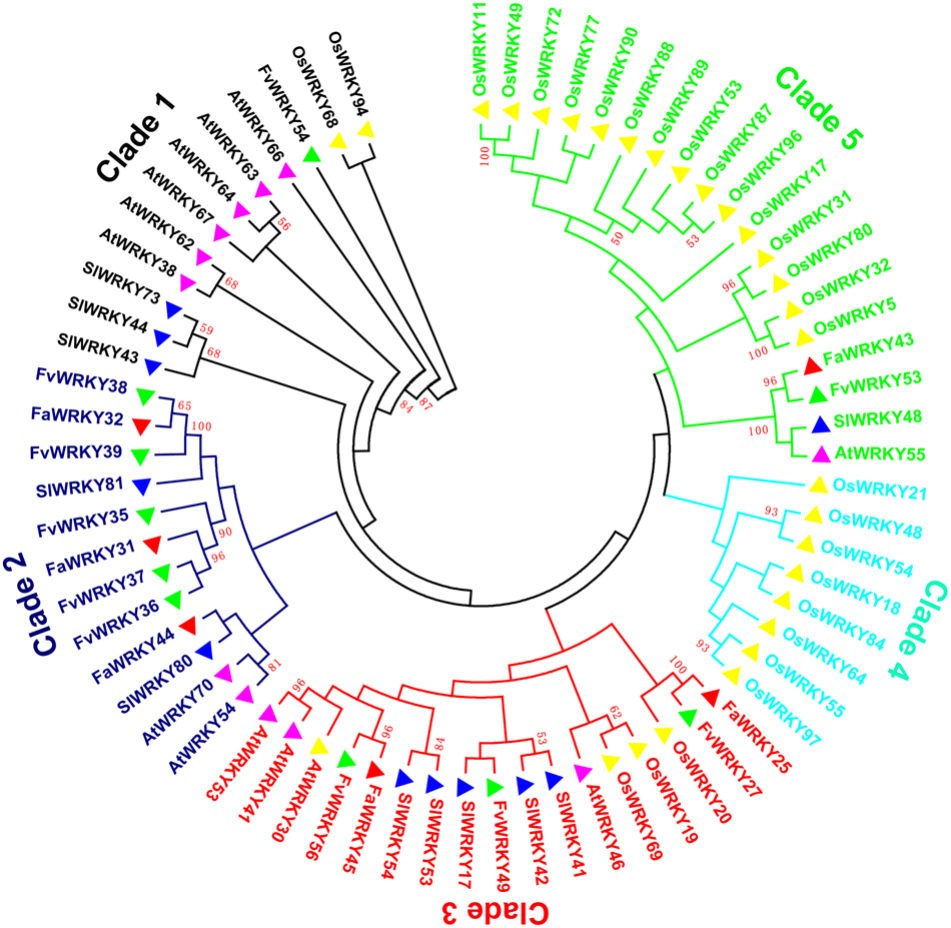
Phylogenetic relationships of group III WRKY proteins from cultivated strawberry, wild strawberry, Arabidopsis, tomato, and rice. The tree was constructed using MEGA 7.0 by the neighbor-joining method. The phylogenetic tree represents 1,000 bootstrap replications. The tree was clustered into five clades (clades 1–6). The different-colored ranges represent different clades. Clade 1 members are indicated in black color; Clade 2 members are indicated in blue color; Clade 3 members are indicated in red color; Clade 4 members are indicated in lighe blue color; Clade 5 members are indicated in pale green color.

The group III WRKY TFs are thought to play a key role in plant evolution and adaptation^19^. To further investigate the phylogenetic relationship of the WRKY III genes in cultivated strawberry, wild strawberry, *Arabidopsis*, tomato, and rice, an unrooted phylogenetic tree of WRKY III complete protein sequences was constructed using MEGA 7.0 (Fig. 4). The phylogenetic tree indicated that the WRKY III proteins were divided into five clades. Clades 5 had the most Group III gene members (19), followed by clade 3 (17), clade 1 (12) and clade 2 (12); clades 4 contained the least WRKY Group III TF members (8) (Fig. 4). Clade 3 and 5 included WRKY Group III TFs in all 5 species, while the members of clades 1 and 2 contained four species. Clade contained other four species except strawberry, while clade 2 only contained all dicots, cultivated strawberry, wild strawberry, *Arabidopsis*, and tomato. Clade 4 was composed of rice only, which might be a monocot-specific clade. Clade 5 consisted of two sub-branch, one was rice, other belong to dicots. This distribution may be related to the split of monocots and dicots. Among those WRKY Group III TFs, six cultivated strawberry WRKY Group III proteins were classified into clade 2 (FaWRKY31, FaWRKY32 and FaWRKY44), clade 3 (FaWRKY25 and FaWRKY45), and clade 5 (FaWRKY43). Five of the six cultivated strawberry WRKY Group III TFs were clustered together with the wild strawberry members. Based on the phylogenetic analysis, five pairs (FaWRKY25 and FvWRKY27, FaWRKY32 and FvWRKY38, FaWRKY43 and FvWRKY53, FaWRKY44 and SlWRKY80 and FaWRKY45 and FvWRKY56) were identified as orthologous genes.

### Expression patterns of cultivated strawberry WRKY III genes in different tissues

**Figure 5:**
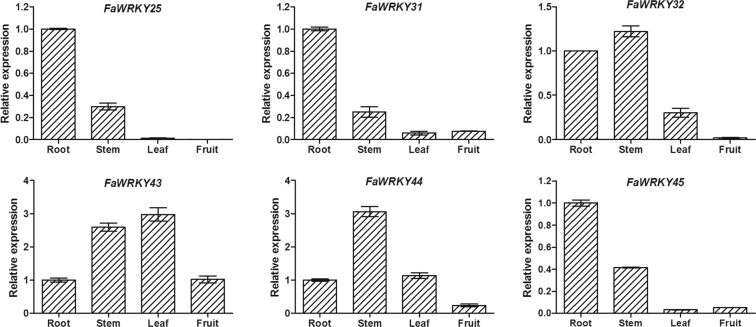
Expression profiles of group III *FaWRKY* genes in different tissues, including roots, stems, leaves and fruits. Bars indicate the standard deviation. Data were normalized to the DNA binding protein (BDP, EU727547) gene using the 2^***−***ΔΔCT^ method.

To obtain insight into the potential functions of FaWRKY group III genes during development, the expression patterns of six FaWRKY group III genes were analyzed in roots, stems, leaves and fruits using qRT-PCR. The six FaWRKY group III genes revealed significant tissue-specific expression patterns (Fig. 5). All six FaWRKY group III genes were expressed in roots and stems. Among the six genes, three showed the highest expression in roots (, *FaWRKY25*, *FaWRKY31* and *FaWRKY45*), two in stems (*FaWRKY32* and *FaWRKY44*), and one in leaves (*FaWRKY43*). Moreover, *FaWRKY25*, *FaWRKY31* and *FaWRKY45* had similar expression patterns and were highly expressed in roots, with little or no expression in the other three tissues.

### Expression profiling of *FaWRKY* genes with RNA-seq in response to continuous cropping

**Figure 6:**
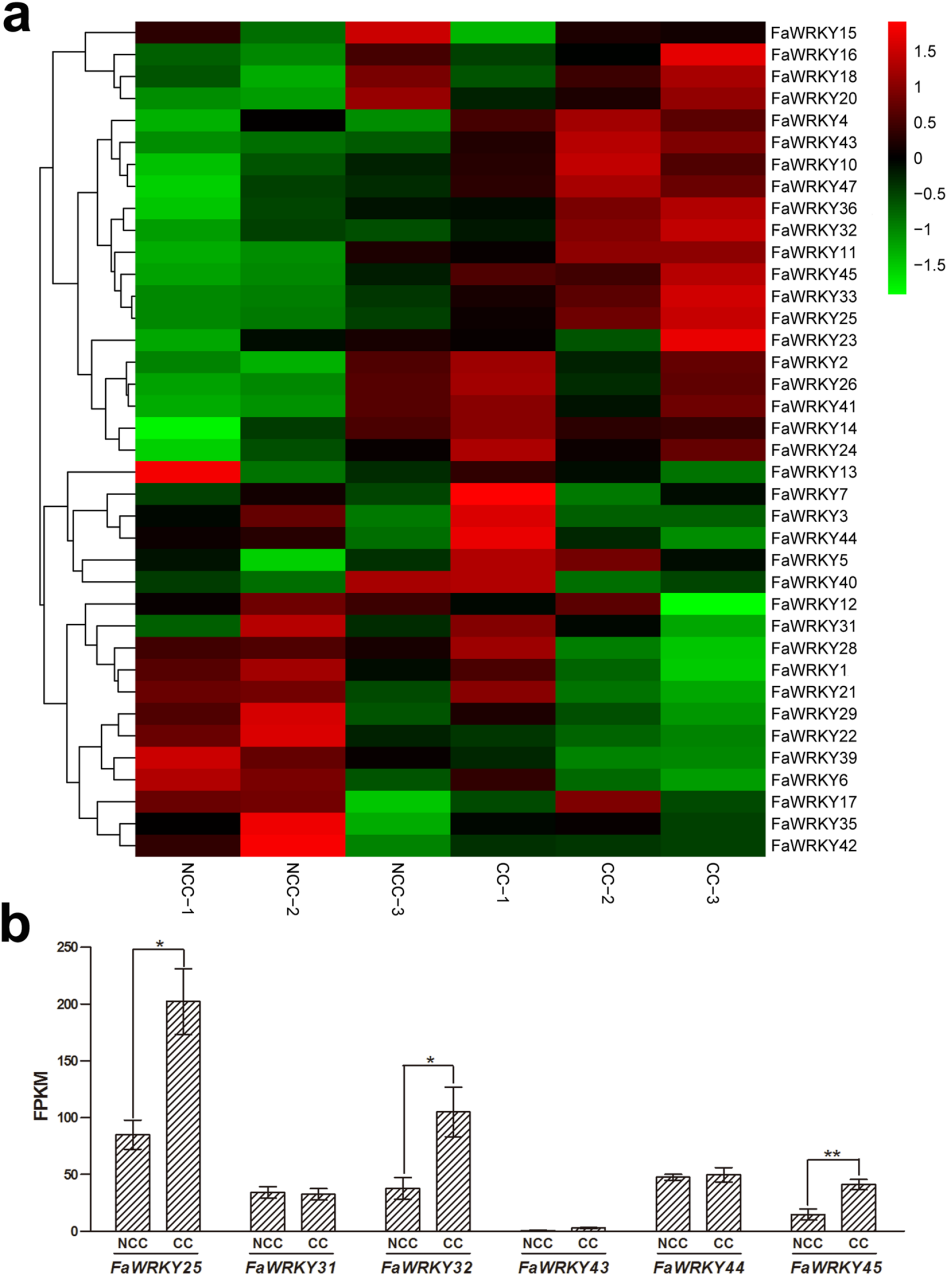
RNA-seq data showing FaWRKY gene expression in response to continuous cropping. (**a**) Heatmap of the expressed *FaWRKY* genes assigned to continuous cropping. The color (from green to red) indicates gene expression intensity from low to high. (**b**) Expression profiles of group III *FaWRKY* genes in response to continuous cropping based on RPKM (reads per kilobase per million mapped reads). *Indicates a significant difference (P < 0.05); **indicates an extremely significant difference (P < 0.01).

In this study, the expression patterns of the *FaWRKY* genes in response to continuous cropping were evaluated based on RNA-seq data. Among the 47 predicted *FaWRKY* genes, 38 showed expression in roots (Fig. 6), and only four *FaWRKY* genes (*FaWRKY25*, *FaWRKY32*, *FaWRKY33* and *FaWRKY45*) changed significantly. The expression of *FaWRKY25*, *FaWRKY32*, *FaWRKY33* and *FaWRKY45* was upregulated in response to continuous cropping (FDR < 0.01 and log2FC ≥ 2). Interestingly, most of the upregulated genes (3/4) belonged to FaWRKY group III. The special expression of group III genes exhibited significant trends in response to continuous cropping. The FPKM of FaWRKY group III genes is shown in Fig. 6b. *FaWRKY45* had significantly higher expression (P-value < 0.01) and *FaWRKY25* and *FaWRKY32* had higher expression (P-value < 0.05) in response to continuous cropping.

### Other changes associated with upregulation of *FaWRKY* genes

**Figure 7:**
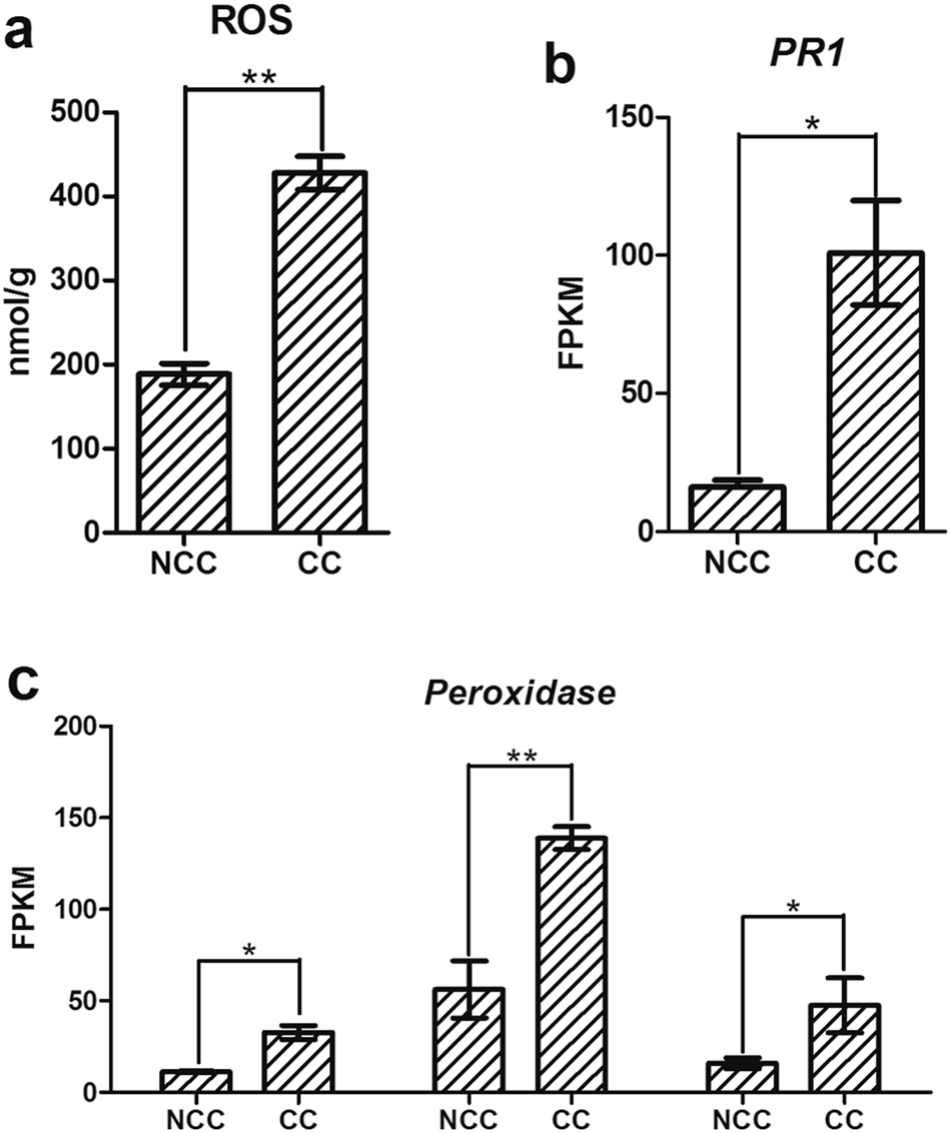
Other changes associated with upregulation of *FaWRKY* genes. (**a**) The level of ROS in NCC roots and CC roots. (**b**) The expression level of *PR1* genes in NCC roots and CC roots; (**c**) the expression levels of *peroxidase* in NCC roots and CC roots.

Reactive oxygen species (ROS) play a vital role in plant–environment interactions. Massive ROS generation is toxic to plant cells and damages cellular membranes. In this study, the level of ROS in continuous cropping roots was significantly higher than that in non-continuous cropping roots (Fig. 7a). The expression level of *PR1* was higher in CC lines than in NCC plants (Fig. 7b). Similarly, the expression levels of *peroxidase* genes were also significantly higher in CC roots than in NCC roots (Fig. 7c).

## Discussion

The WRKY transcription factor genes are involved in the regulation of a large number of processes in plants. Previous studies have revealed some information on the WRKY gene family in many species, such as *Arabidopsis*, rice, tomato, cotton, pineapple and wild strawberry. However, little is known about WRKY gene families in cultivated strawberry.

In the present study, 47 WRKY gene members were identified in cultivated strawberry, and their evolutionary events, conserved structure, stress-related *cis-*elements, comprehensive analysis of group III WRKY genes and expression patterns in continuous cropping were also examined. Distinct expansion in group III WRKY genes was detected in cultivated strawberry under continuous cropping. These results showed that three group III WRKY gene members (*FaWRKY25*, *FaWRKY32* and *FaWRKY45*) were upregulated in response to continuous cropping.

The WRKY gene members were classified into three major groups (I, II and III), and group II was divided into five distinct subgroups (II a–e), as previously described for the model herbaceous plant *Arabidopsis*^9^. The phylogenetic analysis of FaWRKY TFs also indicated that the 47 FaWRKY proteins can be divided into the same groups (I, II a-e, and III). However, *FaWRKY34* could not be clustered into any group, similar to *AtWRKY38* and *AtWRKY52*, which were found in *Arabidopsis*^9^.

The loss of the WRKY domain usually occurs in many monocotyledon species, such as rice and maize^13,20–22^. Group I WRKY TFs in cultivated strawberry all contain two WRKY domains, and no domain loss events were found. The conserved structural domains of their encoded proteins were assessed in this study. Multiple sequence alignments showed that *FaWRKY37* in group II c had unique sequences in the WRKY domain (WRKYGKK). Based on previous studies, sequence variation in the WRKY domain might influence the normal interactions and binding specificities with downstream target genes^15,23,24^.

Stress-related ***cis***-elements were identified in the promoter regions of 47 *FaWRKYs* involved in different functions, such as hormone regulation (ABRE, AuxRR-core, CGTCA motif, P-box, TCA-element, and TGA-element), abiotic stress (LTR and MBS), and disease resistance (TC-rich repeats and W-box elements). The WRKY transcription factors could be regulated by binding different *cis-*elements in their own promoters^25–27^. The WRKY TF promoters could be regulated by autoregulation or cross-regulation by interaction with each other^28,29^. In total, 26 *FaWRKYs* had one or more W-boxes, suggesting that those WRKY TFs might be regulated by autoregulation or cross-regulation.

The group III WRKY gene members might be the most dynamic group with regard to gene family evolution^15,19^. There are 13 group III *WRKY* genes in *Arabidopsis*^30^, 6 in grape^19^, 28 in rice^19^, 10 in *Populus*^19^, and 10 in wild strawberry^14^. However, the number of group III WRKY gene members was related to the diversity of the WRKY gene family size^19^. In this study, group III *FaWRKY* gene members totaled six, which was fewer than the corresponding number in most other plants; this is a potential cause of the smaller number of cultivated strawberry WRKY family members.

The group III gene members play an important role in plant evolution, and their evolutionary history would provide more clues to the origin and evolution of the WRKY gene family^19^. In the current study, the plant WRKY III members were clustered into five clades, and the closely related species might tend to be clustered together. Differences in the orthologous gene pairs between cultivated strawberry/wild strawberry (5) and cultivated strawberry/tomato (1) showed a closer relationship in different strawberries. *FaWRKY44*, which was clustered together with *SlWRKY80*, is not found in wild strawberry.

The expression patterns of WRKY group III genes in different tissues have been evaluated in many species, and there is no uniform gene expression profile for plant WRKY group III genes^15,30^. According to the qRT-PCR expression patterns of all *FaWRKY* group III gene members in different cultivated strawberry tissues, different FaWRKY group III proteins may have diverse functions. In this study, the expression analysis revealed that *FaWRKY25*, *FaWRKY31*, and *FaWRKY45* were highly expressed in the roots, suggesting a putative role in the development of roots. Moreover, those three genes showed similar expression patterns in different tissues, suggesting that these three genes might have retained redundant functions in regulating the same functions^19^.

In plants, WRKY gene members play significant roles in regulating defense gene expression in response to adverse conditions^19^, including biotic and abiotic stresses. Among those WRKY members, the Group III WRKY proteins have been considered to play important roles in regulating plant immunity, resistance and development. For example, the majority of group III members in *Arabidopsis* are involved in pathogen attack and salicylic acid (SA) treatment^30^. The WRKY Group III transcription factors in tomato were identified to participate in the TYLCV defense signaling pathway^25^. Group III *GhWRKY* genes are involved in fiber development and leaf senescence and can be induced by plant hormones, such as jasmonic acid (JA), abscisic acid (ABA), ethylene, and salicylic acid (SA)^6^. Therefore, certain WRKY Group III proteins have significant functions in defense regulation against abiotic and/or biotic stresses.

**Figure 8:**
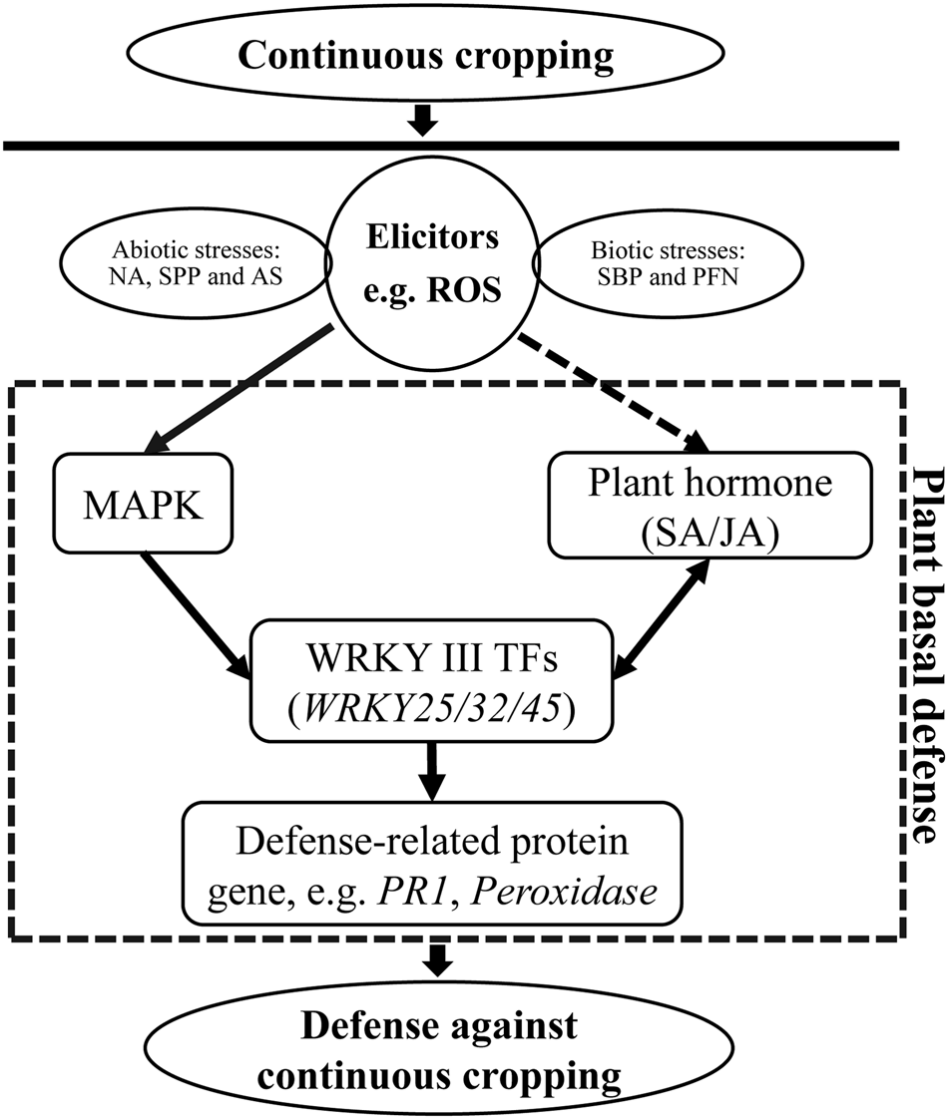
A possible functional network of FaWRKY Group III TFs in response to continuous cropping.

Long-term continuous cropping often brings a complex change in the structure of soil, and strawberry is vulnerable to this problem. The complex stresses to which strawberry responds include biotic stresses, such as the accumulation of soil-borne pathogens and plant-feeding nematodes, and abiotic stresses, such as nutrient availability imbalance, soil physicochemical property deterioration, and autotoxic substance accumulation^31–35^. The ROS network plays a vital role in the signal transduction of resistance to environmental stresses. In plants, WRKYs played essential roles in the ROS scavenging system in plant–environment interactions. During continuous cropping, three FaWRKY Group III gene members (*FaWRKY25*, *FaWRKY32* and *FaWRKY45*) may respond to adverse stress by participating in plant immune ROS bursts by several signaling pathways, such as plant hormone signal transduction and plant-pathogen interactions (Fig. 8). *FaWRKY25*, *FaWRKY32* and *FaWRKY45* have high sequence similarity with *AtWRKY53*, *AtWRKY70* and *AtWRKY41*, respectively. Previous studies showed that *AtWRKY41*, *AtWRKY53* and *AtWRKY70* play important roles in plant basal defense^36,37^. The expression of WRKY is activated by MAPKs and induces the expression of a series of defense-related proteins, such as PR1 protein, peroxidase and plant hormone signaling, similar to salicylic acid (SA) and jasmonic acid (JA)^38,39^. *AtWRKY70* was demonstrated to be involved in regulating SA-JA-mediated signaling pathways, and *AtWRKY41*, *AtWRKY53* and *AtWRKY70* are induced by SA^37,39,40^. The hormone signaling pathway is important in plant immunity and can induce related protein expression to regulate secondary metabolism^25,41^. The extensive cross-regulation mechanism of *FaWRKY25*, *FaWRKY32* and *FaWRKY45*, which belong to FaWRKY Group III, might play an important role in cultivated strawberry continuous cropping defense.

## Materials and Methods

### Data collection

The whole *Fragaria* x *ananassa* annotated genome sequences were obtained from Strawberry GARDEN (http://strawberry-garden.kazusa.or.jp)^42^. The family assignment rules in PlantTFDB (http://planttfdb.cbi.pku.edu.cn/)^43^ were used to identify strawberry WRKY TFs. Finally, the sequences containing WRKY DNA-binding domain (PF03106) were identified as candidates. The annotation of these candidate genes was further checked using BLASTP analysis in NCBI (https://www.ncbi.nlm.nih.gov). The *Arabidopsis thaliana* and *Fragaria vesca* WRKY transcription factor (TF) Group III members were downloaded from the National Center for Biotechnology Information (NCBI, https://www.ncbi.nlm.nih.gov/). The *Oryza sativa* and *Solanum lycopersicum* WRKY TFs were also taken from PlantTFDB.

### Phylogenetic analysis and sequence alignment

The *FaWRKY* genes in cultivated strawberry were classified into different groups based on the *AtWRKY* classification^9^. Multiple alignments of amino acid sequences of FaWRKY proteins were performed by DNAMAN and Clustal X. Multiple sequence alignment for the domain was constructed using the Hidden Markov Model-guided method. MEGA 7.0 was utilized to construct the phylogenetic trees by the neighbor-joining (NJ) method with a bootstrap test of 1000-fold^44,45^.

### Analysis of *cis*-acting elements in *FaWRKY* promoter regions

The upstream sequences (1.5 kb) of the *FaWRKY*-coding sequences were downloaded from Strawberry GARDEN (http://strawberry-garden.kazusa.or.jp)^42^. PlantCARE (http://bioinformatics.psb.ugent.be/webtools/plantcare/html/) was utilized to identify seven regulatory elements of FaWRKYs^46^.

### Plant materials and stress treatments

*Fragaria* × *ananassa* Duch. Benihoppe, a typical cultivated variety, were planted in Beijing Academy of Forestry and Pomology Sciences, Haidian District, Beijing, China (40°1′21″N, 116°16′32″E). All plants were planted in greenhouses at 22 ± 1 °C in a 16 h light/8 h dark photoperiod. The plant materials included two groups: Non-continuous cropping (NCC) and continuous cropping (CC). For NCC strawberry, the plants were cultivated in NCC soil, which cultivated with strawberry for the first time. For CC strawberry, the plants were cultivated in CC soil, which were annually mono-cultivated with strawberry for more than 12 years. The root, stem, leaf and fruit were collected separately for qRT-PCR analysis. Root samples of noncontinuous cropping and continuous cropping treatments were collected at harvest stage and used for further RNA-seq analysis. Each sample contains 3 replicates, and each replicate including 3 plants. All of the collected samples were snap-frozen in liquid nitrogen and kept at −80 °C until further use.

### RNA extraction and gene expression analysis

Total RNA was extracted using the plant RNA Kit (BioTeke, Beijing, China). First-strand cDNA was reverse transcribed using a FastQuant RT Kit (TIANGEN, Beijing, China). Gene-specific primers for qRT-PCR were obtained from qPrimerDB (https://biodb.swu.edu.cn/qprimerdb/) (Table S1)^47^. Real-time PCR was performed using a QuantStudio™ 6 Flex Real-Time PCR System (Applied Biosystems, Foster City, CA, USA) with SYBR® Select Master Mix (Applied Biosystems, Foster City, CA, USA). Each reaction mixture contained 10 μl of 2 × SYBR® Select Master Mix, 1 μl of diluted cDNA product from reverse-transcription PCR, 0.8 μl of each of two primers, and 7 μl of DNase/RNase-free water, and each reaction was repeated using three independent biological and technical replicates. The housekeeping strawberry DNA binding protein (BDP, EU727547) gene was used as an internal control^48^. Gene expression was presented as relative units after standardization using the 2^−ΔΔCT^ method^49^. RNA-seq analysis was performed using an Illumina platform at Beijing BioMarker Corporation. The FaWRKY gene expression levels were estimated by fragments per kilobase of transcript per million fragments mapped (FPKM). The resulting FDR (false discovery rate) was adjusted by the PPDE (posterior probability of being DE). The FDR < 0.01 & |log2(foldchange)| ≥ 2 was set as the threshold for significant differential expression. The heat maps were created by OmicShare tools, a free online platform for data analysis (http://www.omicshare.com/tools).

## Supplementary information

Table S1 The primers of q-PCR.
