# Supplementary material for: Genome-wide characterization of the WRKY gene family in cultivated strawberry (Fragaria × ananassa Duch.) and the importance of several group III members in continuous cropping
Source: Sci Rep. 2019 Jun 10;9:8423. doi: 10.1038/s41598-019-44479-7 (PMC6557897; doi:10.1038/s41598-019-44479-7)
Supplement: Supplementary file 1 — Table S1 The primers of q-PCR. [file 41598_2019_44479_MOESM1_ESM.docx]

**Genome-wide characterization of the WRKY gene family in cultivated strawberry (*Fragaria × ananassa* Duch.) and the importance of several group III members in continuous cropping**

Peng Chen, Qi-zhi Liu^*^

Table S1 The primers of q-PCR.

| Gene |  | Sequences 5'→3' | Annealing Temperature |
| --- | --- | --- | --- |
| *FaWRKY25* | Forward | CCTTTAGGAACACACAAAGCTG | 58℃ |
|  | Reverse | TCTTCCAAATTCTCAGTGTCGA |  |
| *FaWRKY31* | Forward | ATAACGTAATCGGTGGTAGTCC | 58℃ |
|  | Reverse | AAGGGGTGTTCTAGTTCGTAAG |  |
| *FaWRKY32* | Forward | GTACCGAAACGTTTAAGGATCG | 58℃ |
|  | Reverse | AGAATCGTCTTTTGTCCGTACT |  |
| *FaWRKY43* | Forward | GAGGATCATTACATCCAAACGC | 58℃ |
|  | Reverse | ACGGTCAATTTACGAATTTCCC |  |
| *FaWRKY44* | Forward | ATTGTCGATGATTCCCTTCCTT | 58℃ |
|  | Reverse | GCATGTTCGAAACCACATTTTC |  |
| *FaWRKY45* | Forward | AGGACATTCTTGGAGCCAAATA | 58℃ |
|  | Reverse | TGCCTTCCTCGGTAAGTAATTT |  |
